# Dent disease: A window into calcium and phosphate transport

**DOI:** 10.1111/jcmm.14590

**Published:** 2019-08-31

**Authors:** Franca Anglani, Lisa Gianesello, Lada Beara‐Lasic, John Lieske

**Affiliations:** ^1^ Division of Nephrology, Department of Medicine, Laboratory of Histomorphology and Molecular Biology of the Kidney University of Padua Padua Italy; ^2^ Division of Nephrology New York University School of Medicine New York NY USA; ^3^ Division of Nephrology and Hypertension, Department of Medicine, Department of Laboratory Medicine and Pathology Mayo Clinic Rochester MN USA

**Keywords:** ClC‐5, Dent disease, endocytosis, renal calcium transport, renal phosphate transport, sodium/proton exchanger NHE3

## Abstract

This review examines calcium and phosphate transport in the kidney through the lens of the rare X‐linked genetic disorder Dent disease. Dent disease type 1 (DD1) is caused by mutations in the *CLCN5* gene encoding ClC‐5, a Cl^−^/H^+^ antiporter localized to early endosomes of the proximal tubule (PT). Phenotypic features commonly include low molecular weight proteinuria (LMWP), hypercalciuria, focal global sclerosis and chronic kidney disease; calcium nephrolithiasis, nephrocalcinosis and hypophosphatemic rickets are less commonly observed. Although it is not surprising that abnormal endosomal function and recycling in the PT could result in LMWP, it is less clear how ClC‐5 dysfunction disturbs calcium and phosphate metabolism. It is known that the majority of calcium and phosphate transport occurs in PT cells, and PT endocytosis is essential for calcium and phosphorus reabsorption in this nephron segment. Evidence from ClC‐5 KO models suggests that ClC‐5 mediates parathormone endocytosis from tubular fluid. In addition, ClC‐5 dysfunction alters expression of the sodium/proton exchanger NHE3 on the PT apical surface thus altering transcellular sodium movement and hence paracellular calcium reabsorption. A potential role for NHE3 dysfunction in the DD1 phenotype has never been investigated, either in DD models or in patients with DD1, even though patients with DD1 exhibit renal sodium and potassium wasting, especially when exposed to even a low dose of thiazide diuretic. Thus, insights from the rare disease DD1 may inform possible underlying mechanisms for the phenotype of hypercalciuria and idiopathic calcium stones.

## 1 INTRODUCTION

Dent disease (DD) is a rare monogenic, X‐linked recessive disorder characterized by features of incomplete Fanconi syndrome including low molecular weight proteinuria (LMWP). Hypercalciuria is commonly observed, sometimes associated with calcium nephrolithiasis, nephrocalcinosis and/or hypophosphatemic rickets.^1^ Focal global sclerosis and progressive CKD are also common features.^2^ It is unclear whether this global sclerosis and CKD commonly observed in DD are the result of defects at the level of the podocytes or are a result of distal nephron pathology including nephrocalcinosis. Approximately 60% of cases are caused by mutations in the *CLCN5* gene that encodes a Cl^−^/H^+^ antiporter, primarily localized to early endosomes of the proximal tubule (DD1; MIM#300009). Mutations in the *OCRL1* gene, encoding a phosphatidylinositol 4,5‐biphosphate 5‐phosphatase localized at the Golgi apparatus, early endosome and lysosome of proximal tubular cells account for another 15%‐20% of cases (DD2; MIM#300555). The remaining 20% of DD cases remain genetically unresolved.

## 2 ALTERED CALCIUM‐PHOSPHATE METABOLISM IN DENT DISEASE

Most affected males are hypercalciuric, and this can be marked in young children. However, in teenagers and adults the hypercalciuria is typically comparable to that observed in routine calcium stone formers.^3^ Nephrocalcinosis with onset in childhood is common, while nephrolithiasis (calcium oxalate and/or phosphate) occurs in only about 25%. Although hypercalciuria is common, urinary oxalate and citrate excretions are typically normal.

Detailed studies on calcium and phosphate metabolism in DD are few.^4^, ^5^ Blanchard et al^5^ retrospectively analysed 118 genetically confirmed male DD patients (109 DD1 and 9 DD2) and reported that: (a) hypercalciuria was present in almost all patients younger than 3 years old, but calcium excretion declined with age so that the calcium excretion was normal in only 40% of patients under 30 but in 85% of those over the age of 30; (b) mean plasma phosphate was low in all patients, especially after 12 years of age, even with advanced CKD; (c) serum 1,25‐dihydroxyvitamin D [1,25(OH)_2_D] was normal or in the upper range of normal and remained normal even in the late stages of kidney disease; (d) serum 25‐hydroxyvitamin D [25(OH)D] was slightly below normal, and parathormone (PTH) was normal; and (e) plasma potassium decreased with age and hypokalemia was present in 50% patients over the age of 18.

## 3 LOCALIZATION AND FUNCTION OF ClC‐5

ClC‐5 is a 746‐aminoacid protein with multiple membrane‐spanning domains and intracellular N‐ and C‐terminal domain. ClC‐5 belongs to the family of voltage‐gated chloride channels, which is comprised of cell surface channels and intracellular Cl^−^/H^+^ exchangers.^6^

ClC‐5 is highly expressed in the kidney, primarily in the proximal tubular cells (PTCs) of the S3 segment, in the ⍺‐intercalated cells of the cortical collecting duct of mouse and rat kidney, but also in the cortical and medullary thick ascending limb of Henle's loop (mTAL).^7^, ^8^, ^9^, ^10^

**Figure 1:**
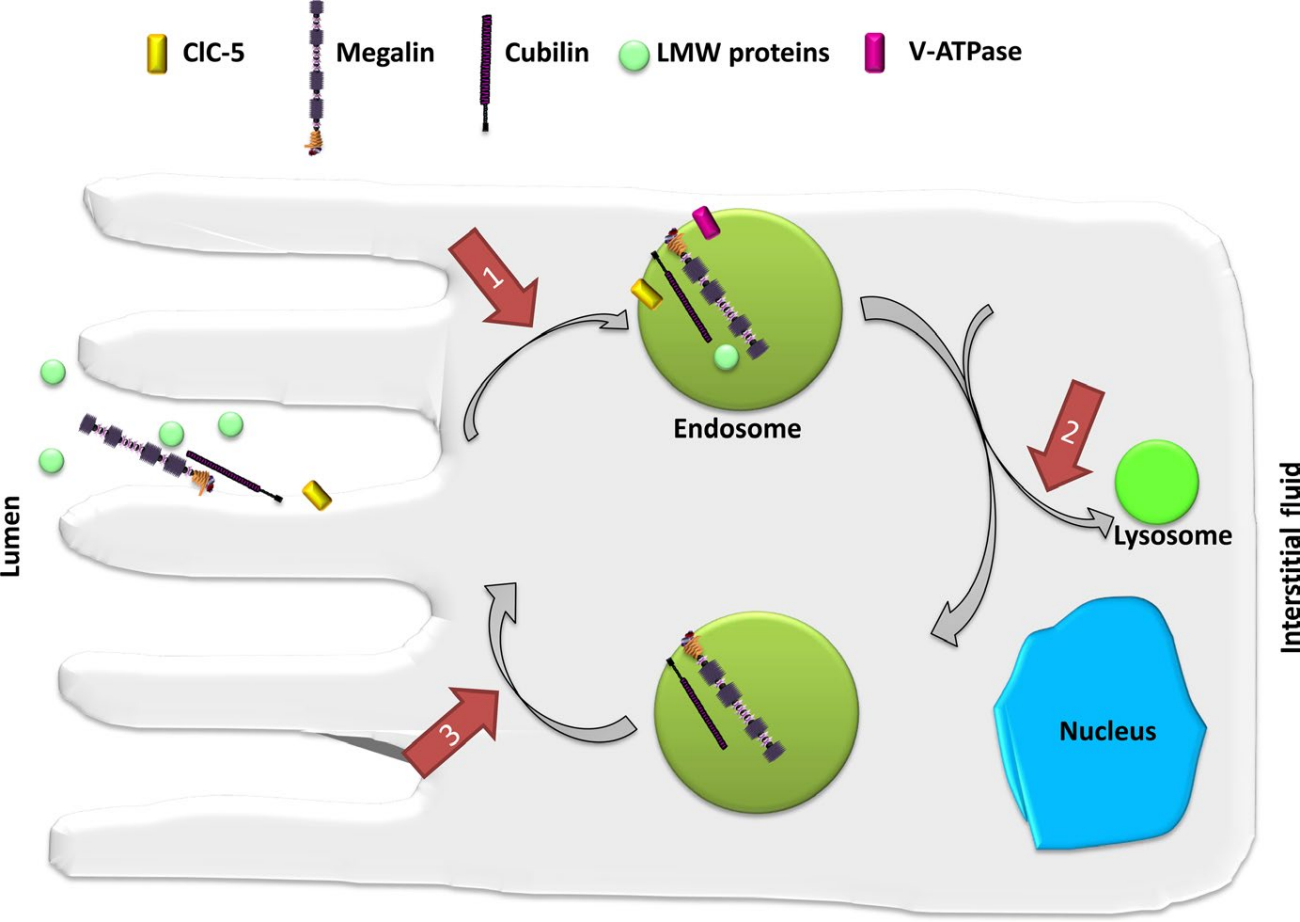
Receptor‐mediated endocytosis of LMW proteins in the proximal tubule. LMW proteins undergo receptor‐mediated endocytosis involving megalin and cubilin. Receptors and ligands are internalized by the formation of endosomes, which are progressively acidified by V‐ATPase. Chloride influx via ClC‐5 facilitates acidification by maintaining electroneutrality of ion transport. Endosomal recycling brings receptors back to the apical surface. The absence or dysfunction of ClC‐5 can potentially disrupt endosome cycling at three points (indicated by arrows): (1) reduced rate of receptor internalization; (2) disrupt progression of endosomes to lysosomes; and (3) disrupt recycling of endosomes to the cell surface

In the PTCs, ClC‐5 plays an important role during receptor‐mediated endocytosis of albumin and LMW proteins that make it through the glomerulus and colocalizes with vacuolar H^+^‐ATPase (V‐ATPase).^11^, ^12^ There ClC‐5 contributes to the acidic pH within the endosomes important for ligand:receptor dissociation and subsequent recycling of the receptor to the apical membrane and degradation of the ligand within endosomes^13^, ^14^ (Figure 1).

The prevalent view was that ClC‐5 supports efficient acidification of endosomes either by providing a Cl^−^ conductance to counterbalance the accumulation of positively charged H^+^ pumped in by V‐ATPase, either directly in parallel with V‐ATPase by acting as a Cl^−^/H^+^ exchanger.^15^ Recently, however, experimental evidence indicates that the endosomal Cl^−^ concentration might play a role in endocytosis independent of endosomal acidification, thus suggesting another possible mechanism by which ClC‐5 dysfunction may cause disease and impair endocytosis.^7^

ClC‐5 may have additional functions since about 8% is located at the PTC plasma membrane.^16^ ClC‐5 plays a key role in the formation/function of the endocytic complex that includes the megalin/cubilin, the sodium‐hydrogen antiporter 3 (NHE3) and the V‐ATPase. Evidence suggests that the large intracellular ClC‐5 C‐terminus plays a crucial function to mediate the assembly, stabilization and disassembly of the endocytic complex via protein‐protein interactions. The Hryciw group demonstrated that the C‐terminus of ClC‐5 also binds the actin‐depolymerizing protein cofilin.^17^ When the nascent endosome forms, recruitment of cofilin by ClC‐5 appears required to localize dissolution of the actin cytoskeleton, thereby allowing the endosome to pass into the cytoplasm. It has been demonstrated both in mouse models and in humans that loss of ClC‐5 causes defective PTC trafficking of megalin and cubilin.^18^, ^19^, ^20^ Thus, ClC‐5 may serve two roles during receptor‐mediated endocytosis: (a) vesicular acidification and receptor recycling; and (b) participation in the non‐selective megalin‐cubilin receptor complex at the apical membrane.

Protein endocytosis in the PTCs relies on active receptors that include not only megalin and cubilin, but also amnionless, disabled‐2.^21^ Megalin and cubilin bind many protein ligands from tubular fluid including the commonly measured LMW proteins β2‐microglobulin, ⍺‐1 microglobulin and retinol‐binding protein (RBP). Whereas megalin binds certain ligands independently, it binds others, such as albumin, jointly with the coreceptor cubilin. Other megalin ligands play key roles in systemic phosphate and calcium metabolism including vitamin D‐binding protein and PTH.

In the collecting duct, ClC‐5 protein localizes to the ⍺‐intercalated cells which are key for acid‐base homeostasis. ClC‐5 colocalizes with the V‐ATPase in the apical and subapical vesicles.^12^ Accordingly, ClC‐5 might be important for insertion and recycling of these vesicles, and when ClC‐5 function is lost, defective expression of V‐ATPase may result causing impaired urinary acidification.

When ClC‐5 expression was silenced by an antisense ClC‐5 transfection in a collecting duct cell model (mIMCD‐3), endocytosis was arrested, and calcium oxalate crystals agglomerated on the ClC‐5 silenced cells, suggesting a possible physiological role for ClC‐5 during crystal clearance in the collecting duct. Thus, loss of ClC‐5 function in this segment may contribute to nephrocalcinosis risk.^22^, ^23^, ^24^ The role of ClC‐5 in mTAL remains circumstantial and speculative.^25^ Studies suggest that endocytosis takes place in murine mTAL where V‐ATPase is also present^26^ albeit at a lesser extent than in PTCs, thus suggesting a possible role for ClC‐5 during endocytosis and exocytosis processes in this nephron segment.

## 4 LOCALIZATION AND FUNCTION OF CALCIUM AND PHOSPHATE TRANSPORTERS IN THE KIDNEY

**Figure 2:**
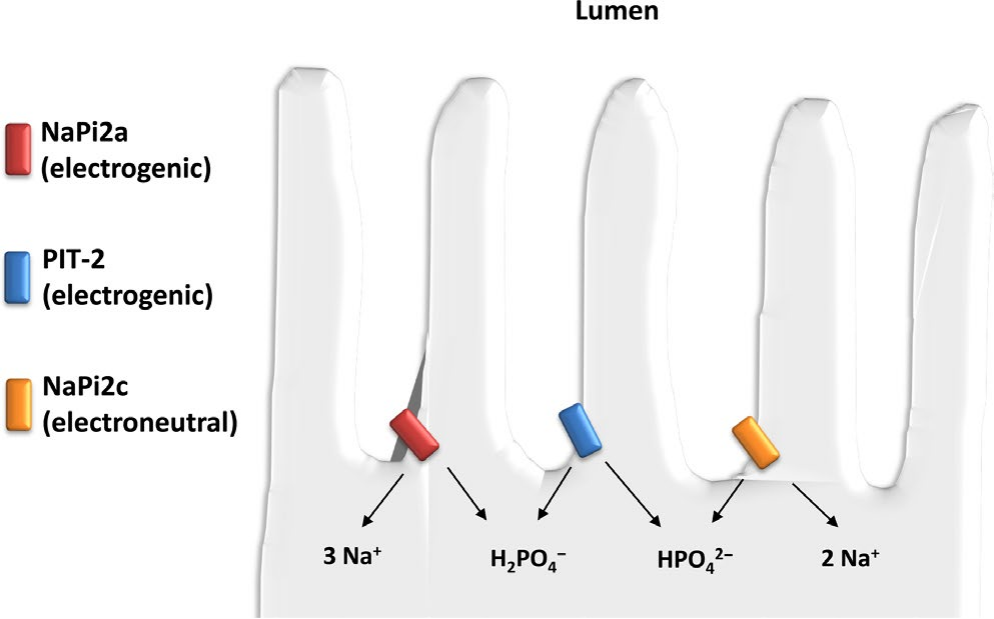
Renal phosphate reabsorption in the proximal tubule. Phosphate is reabsorbed via three sodium phosphate cotransporters: NaPi2a, NaPi2c and PIT‐2. In humans, NaPi2a and NaPi2c are believed to play the most important role in phosphate reabsorption. They are positioned at the apical membrane of renal proximal tubular cells and take advantage of an inward electrochemical gradient for sodium to move phosphate from the filtrate into the cell. The amount of phosphate reabsorbed is dependent on the abundance of the sodium phosphate cotransporters and variations in their number at the brush border membrane are a primary regulatory pathway for urinary phosphate excretion

Approximately 80% of filtered phosphate is reabsorbed from the urine via proximal tubular transporters, mostly in juxtamedullary nephrons^27^, ^28^ (Figure 2).

**Figure 3:**
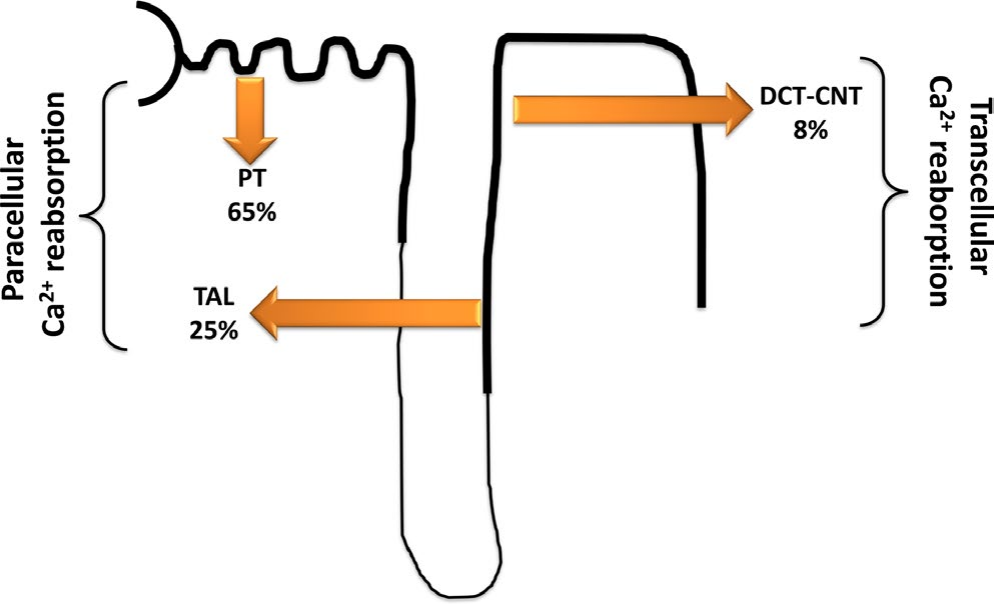
Renal calcium reabsorption along the nephron. The majority of filtered calcium is reabsorbed in the proximal tubule (PT) and thick ascending limb (TAL), with the final highly regulated percentage in the distal convoluted tubule (DCT) and in the connecting tubule (CNT). In the PT, calcium is mainly reabsorbed paracellularly, partially driven by activity of the sodium/proton exchanger 3 (NHE3), which allows transcellular sodium entry at the apical brush border, while the Na‐K‐ATPase pumps sodium out of the cell at the basolateral side. In TAL, calcium is reabsorbed by specialized and controlled paracellular pathways involving claudin 16, 19 and 14. The driving force for calcium is produced by the combined action of the basolateral Na‐K‐ATPase, the Na‐K‐Cl cotransporter (NKCC2) and the outward rectifying ROMK channel on the apical membrane. In DCT‐CNT, calcium enters the cell at the apical side through TRPV5 channels, binds intracellular calbindin‐D‐28k and exits the cell at the basolateral side by the Na‐Ca exchanger (NCX1) and the Ca‐ATPase PMCA4^29^

About 98% of filtered calcium is reabsorbed along the nephron, paracellularly in the PT and in the TAL of the loop of Henle and transcellularly in the distal convoluted tubule and connecting tubule (Figure 3). No reabsorption occurs in the collecting duct; therefore, any calcium delivered there is subject to precipitation depending most importantly upon the tubular fluid pH.^29^ In the PT, the majority of calcium is reabsorbed by passive and hormone‐independent paracellular transport. The claudin family of epithelial tight junction proteins are crucial for paracellular calcium permeability. Active calcium transport in the S3 segment accounts for up to 20%‐30% of PT calcium flux, and however, the precise pathway of this transport is not well defined. Several studies detected apical PT calcium‐sensing receptor (CaSR) expression, a cation‐sensing G protein‐coupled receptor also present in the parathyroid gland.^30^, ^31^ CaSR expression in the PT appears to be regulated by vitamin D, and activation of CaSR may in turn alter PT expression of the vitamin D receptor. Activation of the CaSR in the kidney has other effects on transport, including increased sodium reabsorption and proton secretion in the mouse PT.

Thus, the majority of calcium and phosphate transport localizes in PTCs where ClC‐5 plays an important role in endocytosis. Do these endocytic processes in turn impact PT calcium and phosphate transport?

## 5 PROXIMAL TUBULAR ENDOCYTOSIS AND THE REGULATION OF PHOSPHATE AND CALCIUM HANDLING

### 5.1 Renal phosphate handling

**Figure 4:**
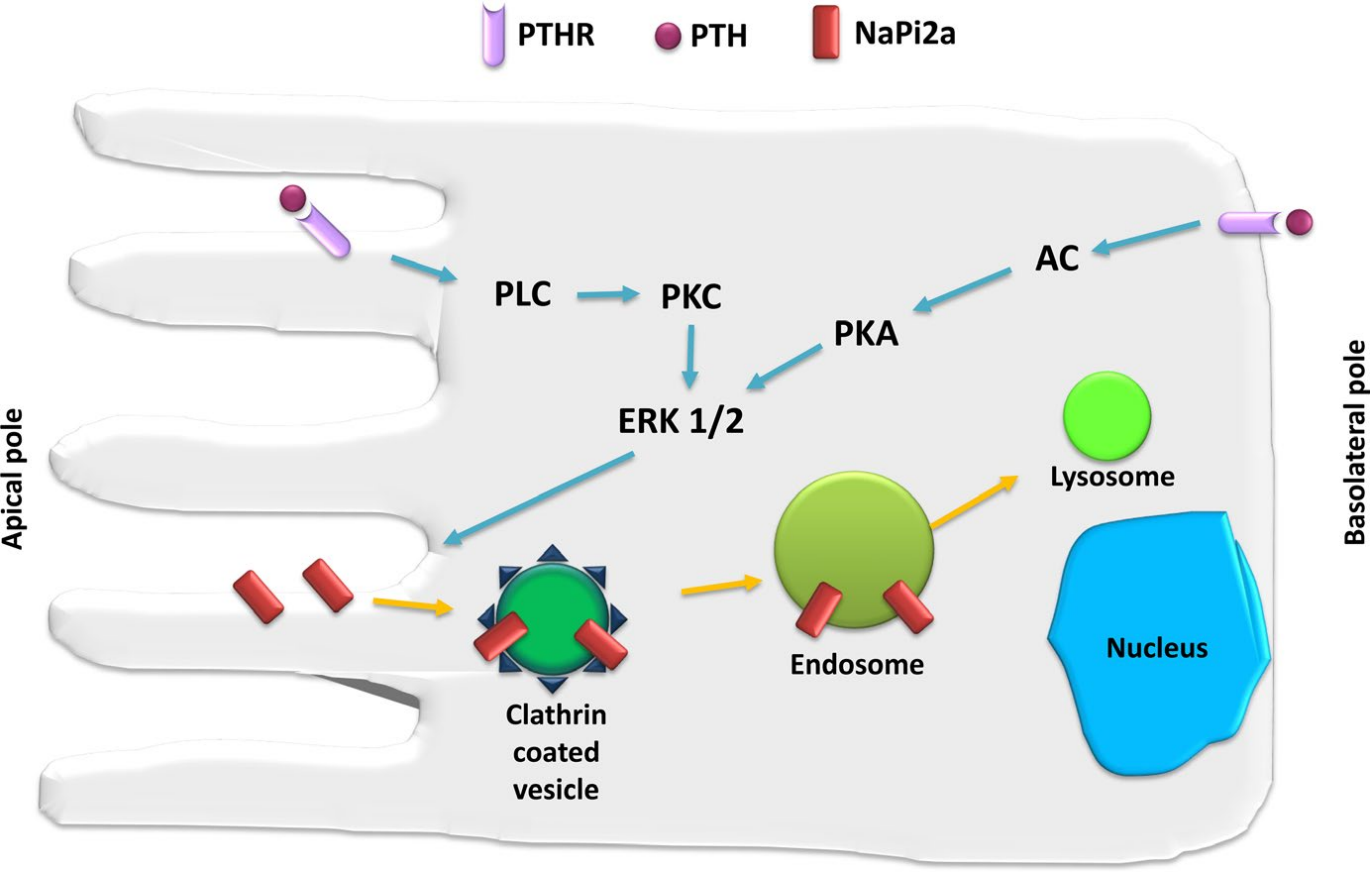
PTH‐induced endocytosis of NaPi2a. In the proximal tubule, PTH binds to apical and basolateral PTH receptors. Stimulation of either receptor is known to rapidly induce phosphaturia by decreasing apical phosphate transporter activity. Activation of phospholipase C (PLC) by apical PTH receptors leads to protein kinase C (PKC)‐dependent stimulation of ERK1/2 kinases and internalization of NaPi2a. Basolateral PTH receptors are linked to adenylate cyclase (AC), protein kinase A (PKA) and ERK1/2. NaPi2a is internalized via clathrin‐coated vesicles, transported to endosomes and targeted to lysosomes for degradation

NaPi2a is the predominant phosphate transporter in the PT.^27^ Major physiological regulators of NaPi2a expression include PTH, dopamine and fibroblast growth factor‐23 (FGF 23), each of which stimulates NaPi2a endocytic removal and degradation as well as decrease mRNA expression.^32^ PTH regulates NaPi2a membrane expression by stimulating NaPi2a endocytosis (Figure 4). This differs from regulation of many other transport proteins that requires modification of the protein itself.^33^

NaPi2a, apical PTH receptors and PLCβ1 are organized in a macromolecular complex via the scaffolding protein Na^+^/H^+^ exchanger regulatory factor (NHERF1).^33^ Phosphorylation of NHERF1 is followed by dissociation of the NaPi2a/NHERF‐1 complex, with NHERF1 remaining at the apical membrane, while NaPi2a is internalized. PTH‐induced inactivation of NaPi2a is facilitated by megalin.^34^ NHERF1 is necessary for PTH‐induced internalization of NaPi2a via apical but not basolateral PTH receptors.^35^ A similar mechanism has been proposed for FGF23 and dopamine regulation of NaPi2a expression.

A low‐phosphate diet has the opposite effect, stimulating insertion of NaPi2a into the apical membrane and inhibiting endocytosis. Because endocytosed cotransporters are degraded in lysosomes, recovery of NaPi2a to basal levels when PTH stimulation is removed depends on de novo synthesis. Thus, apical retention/removal of NaPi2a is a highly regulated process.^33^

Since the scaffold protein NHERF1 plays an important role regulating NaPi2a apical trafficking, as either a chaperone, a scaffolding protein or both,^32^ a link between ClC‐5 and NHERF1 whereby ClC‐5 influences PT apical NaPi2a expression seems possible.

**Figure 5:**
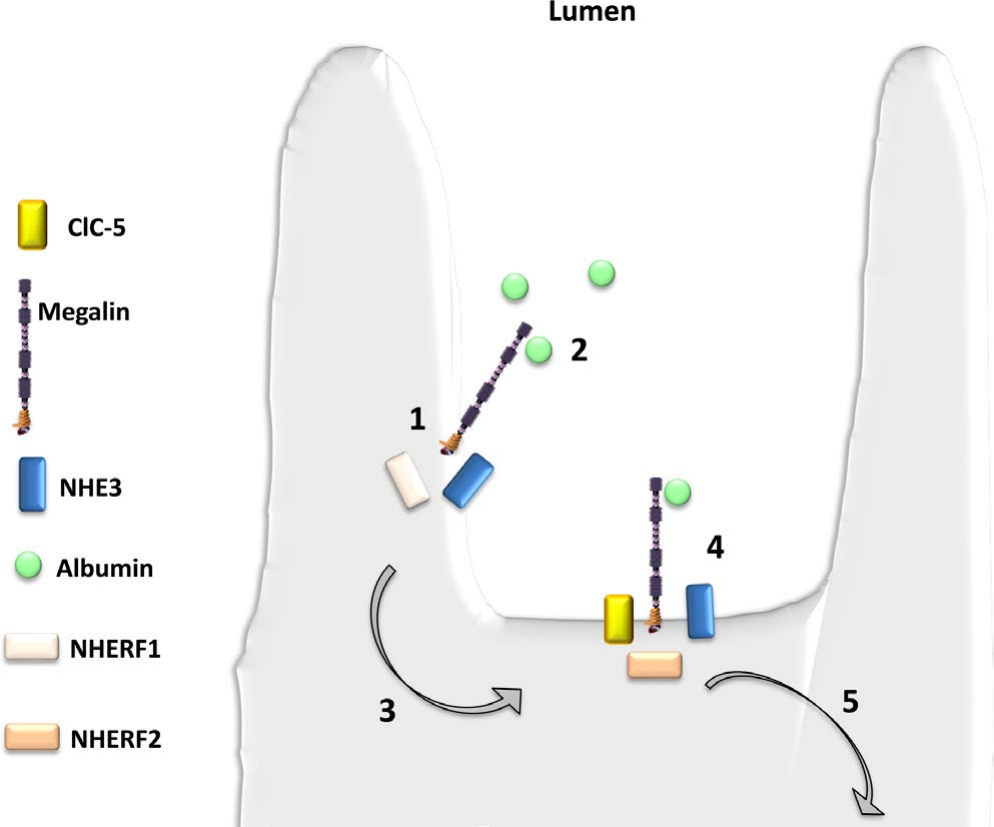
Hypothetical role for NHERF1 in proximal tubular cell endocytosis. NHERF1 could potentially influence proximal tubular endocytosis via the following steps: (1) megalin, via an interaction with NHERF1, associates with NHE3 within microvilli; (2) albumin binds to megalin in the microvilli of proximal tubular cells; (3) the megalin albumin complex translocates to the intravillar cleft; (4) formation of a macromolecular complex containing megalin, NHE3 and ClC‐5 occurs via NHERF2; and (5) the mature endosome is internalized which allows for ligand processing

NHERF scaffold proteins are also crucial for maintaining the macromolecular complex responsible for LMW proteins uptake at the PT brush borders^36^ (Figure 5). Evidence supports this series of events in animal models.^37^, ^38^ The presence of albumin increased levels of plasma membrane‐associated NHERF2, and silencing NHERF2 led to significant reduction in apical membrane ClC‐5, the number of actin clusters and albumin uptake. This in vivo data support a key role for ClC‐5 nucleation of the endocytic complex, presumably by tethering of the complex via NHERF2 to the actin cytoskeleton. These data collectively suggest that NHERF2 is necessary for maintaining ClC‐5 at the plasma membrane. However, the significance of these findings is still not clear, since NHERF1 or NHERF2 KO mice lacked LMWP, the hallmark of ClC‐5 dysfunction.^39^ Intriguingly, however, NHERF1 null mice manifest decreased apical membrane NaPi2a, hyperphosphaturia and hypercalciuria.

### 5.2 Renal calcium handling

**Figure 6:**
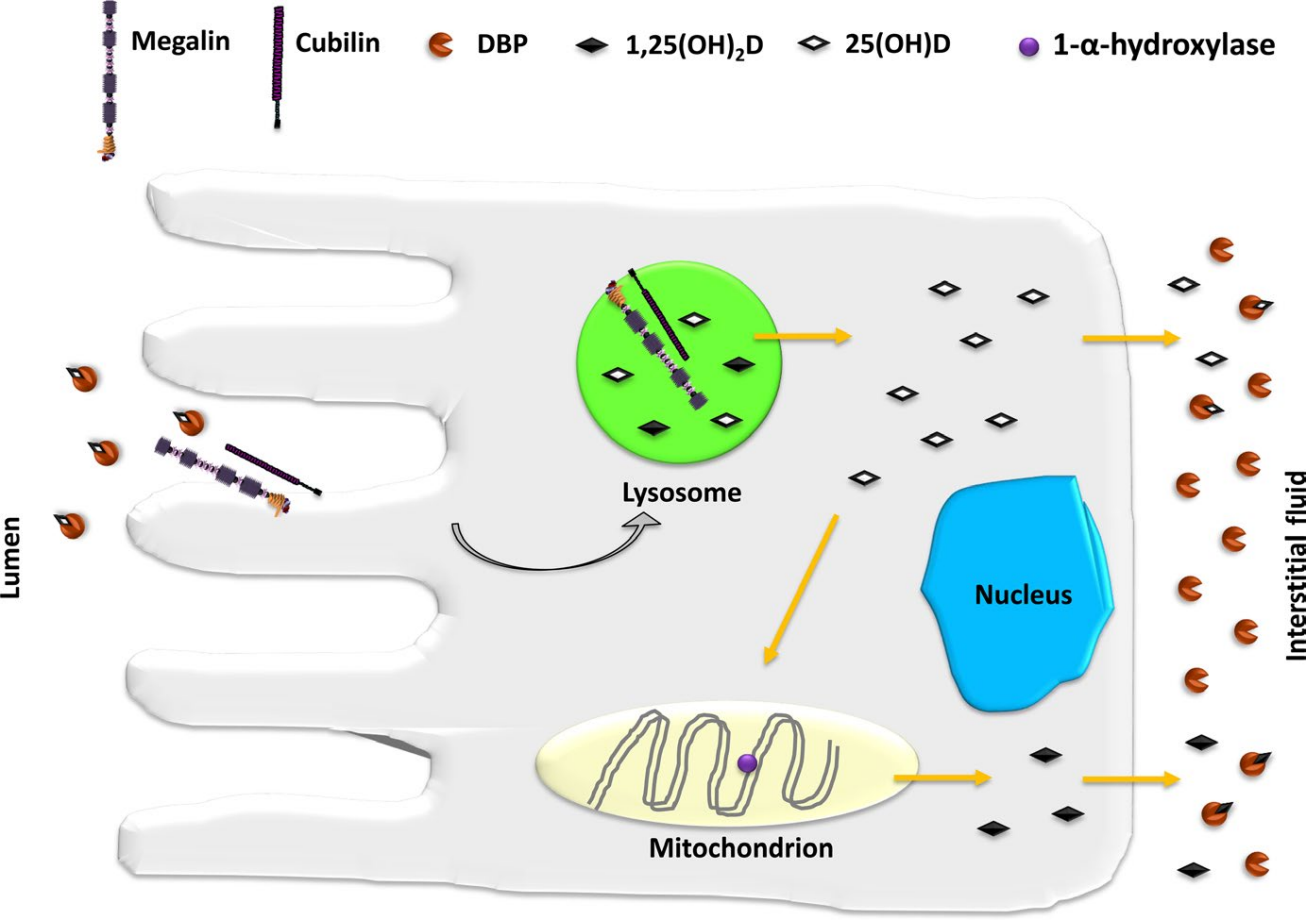
Roles of megalin and cubilin in proximal tubule uptake and activation of 25(OH)D. Vitamin D‐binding protein (DBP) binds 25(OH)D in the circulation, and the complex is freely filtered across the glomerulus to be then reabsorbed in the proximal tubule by receptor‐mediated endocytosis involving megalin and cubilin. The complex is then delivered to lysosomes where DBP is degraded and the vitamin released into the cytosol. 25(OH)D is either secreted directly or trafficked to the mitochondrial membrane where it serves as a substrate for 1‐⍺‐hydroxylase and converted to 1,25(OH)_2_D. 1,25(OH)_2_D is then released into the interstitial fluid where it is complexed by free DBP molecules [modified from reference^40^]

Proximal tubular cells express cytosolic 1‐⍺‐hydroxylase enzyme and are the major source of circulating active 1,25(OH)_2_D (Figure 6). These cells also contain 24‐hydroxylase that converts 1,25(OH)_2_D to the biologically inactive metabolite calcitroic acid.^40^, ^41^ PTCs sense circulating 1,25(OH)_2_D to modulate activity of the 1‐⍺‐hydroxylase and 24‐hydroxylase. Wang et al^42^ demonstrated that in the absence of vitamin D, the vitamin D receptor (VDR) localizes to the interior face of the apical brush border. When 1,25(OH)_2_D is administered to vitamin D‐deficient mice, VDR bound to 1,25(OH)_2_D translocates from the brush border to the cytoplasm and nucleus where it suppresses 1‐⍺ ‐hydroxylase and stimulates 24‐hydroxylase.

Parathormone, a key modulator of both renal calcium and phosphate handling, is freely filtered by the glomerulus and reaches the luminal surface of PTCs where it can bind the PTH receptor or instead be reabsorbed via megalin‐mediated endocytosis. Biochemical and in vitro studies demonstrated that PTH regulates 1,25(OH)_2_D synthesis via G protein signalling.^43^ A recent study revealed that the ⍺‐subunit of the stimulatory G protein (Gsα) is essential for PTH action in the PT and that the loss of this signalling protein results in PTH resistance, leading to hypocalcemia, reduced serum 1,25(OH)_2_D and elevated serum PTH. The Gsα KO mice also demonstrated significantly elevated PT 24‐hydroxylase mRNA.^44^

In a rat model of 1,25(OH)_2_D deficiency, Silva et al^45^ observed that 1,25(OH)_2_D deficient and parathyroidectomized rats had low levels of cortical ClC‐5 mRNA and protein, and lower serum and higher urinary calcium concentrations compared with control and 1,25(OH)_2_D‐deficient rats. Administration of PTH increased ClC‐5 mRNA and protein expression to near‐normal levels and decreased renal calcium leak. The authors concluded that PTH modulates cortical ClC‐5 levels at both the mRNA and the protein level, but not in the medulla, suggesting that PTH, which is known to be a major mediator of calcium reabsorption in the kidney cortex, may act via regulation of ClC‐5. They speculated that PTH could regulate ClC‐5 expression in order to maintain the number of vesicles able to recycle membrane and endocytosed proteins, including NaPi2a.

Together, these data strongly support a pivotal role for PT endocytosis in renal calcium and phosphorus handling.

## 6 IN VIVO ClC‐5 KO MODELS: IMPACT ON PHOSPHATE AND CALCIUM TRANSPORT MECHANISMS

### 6.1 Phosphate transport

Studies using two independent ClC‐5 KO mice strains, the so‐called Jentsch^46^, ^47^ and Guggino models,^18^, ^48^, ^49^, ^50^ provided critical insights into the mechanisms of PT dysfunction in DD1. These two strains recapitulate the major features of DD1 including LMWP. Studies in the two models demonstrated that ClC‐5 inactivation is associated with severe impairment of both fluid‐phase and receptor‐mediated endocytosis, and a trafficking defect with the loss of megalin and cubilin at the PT brush border (Table 1). However, targeted disruption of ClC‐5 in the Jentsch model did not lead to hypercalciuria, kidney stones or nephrocalcinosis, while a similar disruption in the Guggino model did.^48^ Mice in the Jentsch model produced slightly more acidic urines. Urinary phosphate excretion was increased in both models by about 50%. Hyperphosphaturia in the Jentsch model was associated with decreased apical NaPi2a expression, although principally at the brush border of the S1 PT segment and at subapical vesicles of other PT segments. This was surprising because defective NaPi2a endocytosis due to loss of ClC‐5 function might be expected to result in increased plasma membrane presence. Indeed, observed changes in NaPi2a surface expression in these KO mice appeared ClC‐5‐independent since apical NaPi2a was not decreased in any PTs of chimeric female mice, while it was in all PTs of ‐/y male mice.

**Table 1:** Comparing phenotypes and molecular players in the Jentsch and Guggino mouse models

	Jentsch model	Guggino model
ClC‐5 KO method	Targeting part of exon 5 and 6; C57BL/6 strain	Targeting exon 6 C57BL/6 strain
Renal phenotype		
Low molecular weight proteinuria	Vitamin D‐binding protein, retinol‐binding protein	Vitamin D‐binding protein, Clara cell protein of 16 kDa, transferrin
Defective receptor‐mediated endocytosis	β2‐microglobulin, lactoglobulin	β2‐microglobulin
Megalin	Not tested	Defective trafficking
Cubilin	Not tested	Defective trafficking
Defective fluid‐phase endocytosis	Fluorescein isothiocyanate‐dextran, horseradish peroxidase	Fluorescein isothiocyanate‐dextran, horseradish peroxidase
Polyuria	Present	Present
Sodiuria	Not tested	Increased
NHE3	Decreased apical exposure	Decreased protein abundance
Phosphaturia	Present (decrease apical exposure of NaPi2a)	Present
Hypercalciuria	Absent	Present
Glycosuria	Not reported	Present
Amino‐aciduria	Not reported	Present
Bone and mineral phenotype		
Nephrocalcinosis	Absent	Intrarenal calcium deposits von Kossa positive
Serum 1,25(OH)2D	Reduced	Increased
Serum 25(OH)D	Reduced	Not tested
Serum PTH	Normal	Normal
Urine 1,25(OH)2D	Increased	Not tested
Urine 25(OH)D	Increased	Not tested
Urine PTH	Increased	Not tested
TRPV5	Up‐regulated	Not tested
Bone turnover markers	Not tested	Increased

Depriving ClC‐5 ‐/y mice of dietary Pi enhances plasma membrane NaPi2a expression to normal levels. When these KO mice were given PTH, NaPi2a was internalized but at a slower rate compared with WT mice. The reduced internalization of NaPi2a in response to PTH suggests that altered PTH delivery might lead to vesicular localization of NaPi2a in the S3 PT segment of KO mice. Indeed, whereas serum PTH is normal in KO mice, urinary PTH is increased by about 1.7‐fold. Because megalin is down‐regulated in these mice, luminal PTH levels are increased. The increased stimulation of apical PTH receptor in turn enhances internalization of NaPi2a in more distal segments of the PT.

In conclusion, the phosphaturia in KO animals may be a consequence of reduced endocytosis of filtered PTH.

### 6.2 Calcium transport

The possibility that ClC‐5 may participate in renal calcium transport was suggested by the finding that ClC‐5 expression in the renal cortex is under PTH regulation and that renal ClC‐5 expression inversely correlated with urinary calcium excretion.^46^ The hypothesis that hypercalciuria in DD1 is intrinsic to kidney‐mediated mechanisms is supported by the observation that hypercalciuria is absent after kidney transplantation.^3^, ^51^ However, other reports have raised the possibility that ClC‐5 may be linked to gastrointestinal calcium absorption and that hypercalciuria observed in DD1 may represent a secondary event.^4^

The most striking difference between the Jentsch and Guggino models is the absence of hypercalciuria, nephrocalcinosis and nephrolithiasis in the Jentsch model. However, the Jentsch model strongly supports a role for endocytosis in renal PT calcium handling. Increased tubular PTH concentrations due to a lack of ClC‐5‐related endocytosis should stimulate the PTH receptor and induce up‐regulation of 1‐α‐hydroxylase. The amount of 1‐α‐ hydroxylase mRNA is in fact increased in ClC‐5 KO mice. Assuming that 25(OH)D is unchanged, elevated 1,25(OH)_2_D levels would be expected, as is observed in ClC‐5 KO mice and patients with DD1. Increased 1,25(OH)_2_D should enhance intestinal calcium absorption leading to hypercalciuria and kidney stones. However, uptake of 25(OH)D via apical endocytosis is also defective in ClC‐5 KO mice. In fact, a great quantity of the 25(OH)D appears in the final urine. Thus, a delicate balance between reduced 25(OH)D supply and stimulation of the 1⍺‐hydroxylase, both of which result from a lack of ClC‐5 dependent endocytosis, will determine the presence or absence of hypercalciuria. The ultimate outcome may depend on nutritional or genetic factors.

The hypothesis that hypercalciuria in mice with defective ClC‐5 function is in fact due to intestinal hyperabsorption of calcium rather than a renal leak was supported by another ClC‐5 KO mouse model.^52^ By targeting ClC‐5 expression with antisense ribozyme, the authors demonstrated that hypercalciuria was abolished by calcium deprivation, suggesting that intestinal calcium hyperabsorption was important.

The Jentsch group studied vitamin D metabolism in their ClC‐5 KO mouse model in depth using expression profiling, qRT‐PCR and hormone measurements.^53^ They demonstrated a large increase in 1‐α‐hydroxylase in parallel with a decrease in 24‐hydroxylase. Although the up‐regulation of 1‐α‐hydroxylase and down‐regulation of 24‐hydroxylase would be predicted to increase formation of 1,25(OH)_2_D, the concentration of this active metabolite was instead reduced in the serum of ClC‐5 KO mice. However, target genes of 1,25(OH)_2_D were up‐regulated in KO kidneys. Expression analysis of intestine and bone revealed that the up‐regulation of 1,25(OH)_2_D target genes was not systemic but kidney intrinsic. In spite of reduced serum levels of 1,25(OH)_2_D in ClC‐5 KO mice, 1,25(OH)_2_D was increased in later nephron segments as a consequence of impaired PT endocytosis. These changes could be attributed to impaired endocytosis due to lack of ClC‐5 function. Other target genes of 1,25(OH)_2_D were up‐regulated in the distal nephron segments. In this model, the authors demonstrated that this up‐regulation was kidney specific, that is the effect of 1,25(OH)_2_D on distal vs proximal tubules was local and direct. The renal distal up‐regulation of the calbindins and TRPV5 through VDR is predicted to increase renal calcium reabsorption, consistent with the lack of hypercalciuria in their mouse strain.

The results obtained from studies using the Guggino model^49^ are in marked contrast to the results from the Jentsch model. In the Guggino model, a small number of mice had deformities of the dorsal spin and backward growth of teeth but not osteopenia or rickets. Moreover, Guggino mice had more than doubled urine calcium excretions and developed nephrocalcinosis at the corticomedullary junction. In this model, hypercalciuria on low‐ and high‐calcium diets is of bone and renal origin, and is not caused by increased intestinal calcium absorption, despite the elevated level of serum calcitriol.

The discrepancy between the two mouse models regarding mechanisms of hypercalciuria in DD1 might suggest another possibility, that is it might be caused by defective NHE3 expression at the apical PT. A link between sodium and calcium reabsorption is well known. Thiazide diuretics that block the Na^+^‐Cl^−^ cotransporter (NCC) in the distal convoluted tubule have a hypocalciuric effect stimulating sodium and calcium reabsorption in the PT.^54^, ^55^ The effect of diuretics on renal tubular calcium transport has been recently reviewed by Alexander and Dimke,^56^ and studies suggest the PT as the major site of calcium reabsorption strictly linked to sodium reabsorption.^57^

## 7 THE Na^+^/H^+^ EXCHANGER NHE3 AND CALCIUM REGULATION AT THE PROXIMAL TUBULE

**Figure 7:**
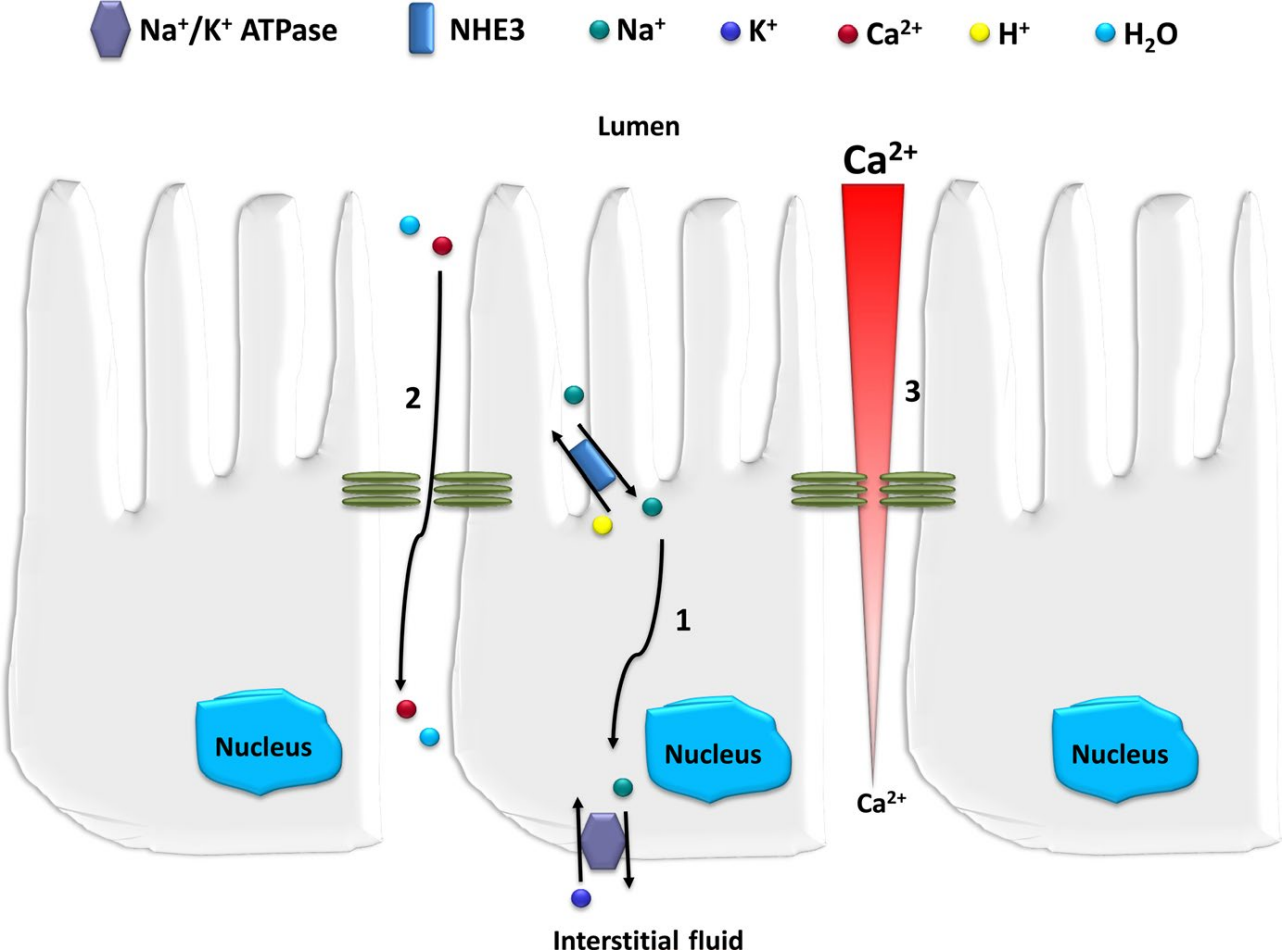
Role of NHE3 in paracellular Ca^2+^ reabsorption in the proximal tubule. NHE3 is responsible for the majority of Na^+^ and water reabsorption from the proximal tubule. In exchange for a luminal H^+^, NHE3 mediates the influx of Na^+^ into the proximal tubular cell. This exchange is driven by an inward electrochemical gradient for Na^+^, driven by basolateral Na^+^/K^+^ATPase (1). This active transcellular Na^+^ flux creates the osmotic driving force for water reabsorption, which, in turn, drives paracellular Ca^2+^ reabsorption (2) either by convection/solvent drag or by creating a concentration gradient for Ca^2+^ (3) [modified from reference^57^]

The Na^+^/H^+^ exchanger (NHE) protein family contains at least nine isoforms divided into plasma membrane isoforms (NHE1‐NHE5) and endomembrane isoforms (NHE6‐NHE9).^58^ The plasma membrane isoforms function to exchange extracellular Na^+^ for intracellular H^+^. In adult kidney, PT NHE3 is the predominant brush border Na^+^/H^+^ exchanger (Figure 7).

Recent studies highlight the importance of PT NHE3 for calcium reabsorption. NHE3^−/−^ KO mice manifest significant urinary calcium wasting.^59^ Plasma Ca^2+^ was not different between WT and NHE3^−/−^ KO mice was nor plasma PTH. However, 1,25(OH)_2_D was dramatically elevated in these mice, and despite this, intestinal Ca^2+^ absorption was reduced. The reduced intestinal Ca^2+^ absorption and increased urinary calcium excretion occurred at the expense of bone health. In fact, NHE3^−/−^ KO mice demonstrated decreased cortical bone mineral density and trabecular bone mass. When NHE3 was overexpressed in opossum kidney cells, the paracellular calcium flux across confluent monolayers was increased. The authors proposed that the predominant mechanism mediating these observations was the absence of a driving force generated by NHE3 that reduced intestinal and proximal tubular paracellular calcium flux.

The *SLC9A3* gene encodes human NHE3, and *SLC9A3* homozygous or compound heterozygous disease‐causing mutations have recently been reported in 9 patients from 8 families with congenital secretory sodium diarrhoea (MIM#616868).^60^, ^61^ Variants in the *SLC9A3* gene influence susceptibility to bacterial infections and severity of lung disease by interacting with the *CFTR* gene, providing evidence for a potential modulatory effect in Cystic Fibrosis.^62^, ^63^ No association has been found to date between *SLC9A3* variants and renal proximal tubulopathies.

Sodium‐hydrogen antiporter 3 is a highly regulated protein that is directly or indirectly influenced by a variety of factors.^64^, ^65^ PTH is known to regulate Na^+^ transport in PT. Animal and cell culture studies have shown that PTH inhibits NHE3 activity and expression, acutely and chronically, by reducing total and apical protein levels, as well as *NHE3* mRNA abundance.^66^, ^67^ Thus, besides exerting a hypercalciuric effect by stimulating 1,25(OH)_2_D synthesis, PTH might indirectly favour a renal calcium leak by inhibiting NHE3. Perhaps counterbalancing these effects, PTH also stimulates calcium transcellular reabsorption in distal convolute tubules via the epithelial calcium channels TRPV5 and TRPV6, independent of 1,25(OH)_2_D.^68^

Very recently, Edwards and Bonny^69^ examined PT Ca^2+^ reabsorption in relation to Na^+^ and water influx and PTH by expanding a previously published mathematical model of water and solute transport in the rat PT. Their results indicated that PT Ca^2+^ reabsorption is principally driven by a transepithelial Ca^2+^ concentration gradient that stems from water reabsorption which is itself coupled to Na^+^ reabsorption. The model predicts that PTH‐mediated inhibition of the apical NHE3 reduces Na^+^ and Ca^2+^ transport to a similar extent. Furthermore, PTH is predicted to exert and indirect impact on PO_4_ reabsorption via its inhibitory action on NHE3.

From these studies, the importance of PT NHE3 regulation clearly emerges, not only on calcium but also on phosphate handling.

## 8 ClC‐5 KO MODELS: WHAT CAN THEY TELL US ON NHE3 REGULATION?

Sodium‐hydrogen antiporter 3 is dysregulated in ClC‐5 KO mice. In the Jentsch model,^46^ PT NHE3 expression was reduced to a similar degree as NaPi2a, and however, dietary phosphate deprivation resulted in increased apical expression. PTH‐induced endocytosis of NHE3 was also markedly reduced in the KO mice. Thus, vesicular localization of NHE3 appeared mediated by ClC‐5‐dependent defective PTH endocytosis. The decreased activity of NHE3 may be the cause of polyuria observed in these mice.

Decreased PT NHE3 protein abundance was also observed in the Guggino ClC‐5 KO model. Using transport activity measurement assessed via two‐photon microscopy, they demonstrated reduced basal activity of NHE3.^70^ Moreover, ClC‐5 inhibition by shRNAi in opossum kidney cells resulted in reduced expression of both megalin and NHE3. However, it is not currently possible to assess direct effects of ClC‐5 on NHE3 trafficking. In fact, NHE3 is a part of a macromolecular complex required for LMW protein receptor‐mediated endocytosis.^16^, ^17^, ^71^ Likewise, NHE3 appears to play a crucial role in PT receptor‐mediated endocytosis.^72^ In these in vitro studies, NHE3 inhibition dramatically reduced PT endocytosis and alkalinized the early endosome. Results were further confirmed in NHE3 KO mice to demonstrate that urinary protein excretion was significantly higher in mutant compared with WT mice. The urinary protein patterns resembled that of megalin or ClC‐5‐deficient mice, that is typical models of tubular proteinuria. Thus, PT deficiency of NHE3 leads to reduced protein reabsorption.

Blockade of NHE3 activity by an NHE3 inhibitor in ClC‐5‐depleted opossum kidney cells reduced endocytosis to 46% of control levels.^17^ In ClC‐5 KO cells, the inhibitor still effectively decreased endocytosis to 56% of that in the untreated KO cells. This study further confirms the contribution of NHE3 to endocytosis and to acidification of the early endosomal compartment.

In ClC‐5 KO opossum kidney cells, surface NHE3 expression was reduced despite an unchanged total protein level.^70^ PTH decreased NHE3 surface expression, but the extent of the decrease and the rate of endocytosis were not different from control cells. Conversely, the rates of basal and dexamethasone‐stimulated exocytosis of NHE3 were diminished in ClC‐5 KO cells. The authors concluded that ClC‐5 is necessary for NHE3 exocytosis and offers an alternative hypothesis regarding reduced NHE3 expression in ClC‐5 KO mice.

Collectively, these studies highlight not only the crucial role of NHE3 in renal calcium handling and the importance of ClC‐5 in regulation of NHE3 trafficking, but also the role of NHE3 in PT receptor‐mediated endocytosis.

## 9 SODIUM AND WATER WASTING IN DENT DISEASE PATIENTS

Besides reduced PT NHE3 expression in ClC‐5 KO mice, ClC‐5 impairment could be hypothesized to cause defects in salt reabsorption not only in PT but also in more distal segments. A defect in mTAL, where ClC‐5 is expressed, could impact tubulo‐glomerular feedback and potentiate the salt reabsorption defect. A Bartter‐like syndrome has also been occasionally reported in patients with DD1.^73^, ^74^, ^75^, ^76^ Bartter‐Gitelman and hyperprostaglandin E syndromes, inherited disorders that manifest hypokalemic metabolic alkalosis, are caused by malfunction of renal tubular electrolyte transporters or ion channels including ClC‐K‐type chloride channels.^77^ All variants share several clinical characteristics, including renal salt wasting, hypokalemic metabolic alkalosis and hyper‐reninemic hyperaldosteronism.

Wojciechowski et al^78^ very recently reported that barttin, the protein impaired in Bartter syndrome type 4, is an essential subunit for the ClC‐Ka and ClC‐Kb chloride channels and appears to regulate subcellular localization and post‐translational modification of ClC‐5. The functional characterizations of the ClC‐5 mutant G261E that has been associated with Bartter‐like symptoms demonstrated the increased abundance in the ER that could reduce the ClC‐K trafficking to the surface membrane and produce the atypical symptoms observed in the affected patients. Based on these results, they hypothesized that abnormal Barttin‐ClC‐5 interactions in the TAL or CD could contribute to the Bartter phenotype observed in some patients with DD1.

Blanchart et al^5^ found that about 50% of patients with DD1 older than 18 years of age presented with hypokalemia without changes in serum bicarbonate. Renal potassium loss was explored in six hypokalemic patients with DD1. All had inappropriate kaliuresis and low‐range blood pressure, with a high renin concentration. Plasma aldosterone concentration was high in some patients and normal in others owing to a combination of the stimulatory effect of renin and the inhibitory effect of hypokalemia on aldosterone production. Four patients had marked polyuria. These data reveal that hypokalemia is an important trait of DD1 phenotype, at least in adult patients.

## 10 CONCLUSIONS

Observations in patients with DD1 and model systems highlight the importance of receptor‐mediated endocytosis for PT function. The PT plays a pivotal role in renal phosphate and calcium handling, exerted mainly through the regulation of ClC‐5‐dependent (PTH) endocytosis. In fact, reduced expression of megalin, by mediating the uptake of PTH, antagonizes the effect of increased PTH availability on the luminal PTH receptor. Thus, the delicate balance between PTH reabsorption and PTH receptor activation influences both NaPi2‐mediated proximal tubular phosphate reabsorption and the highly regulated proximal tubular synthesis of vitamin D.

ClC‐5‐dependent PTH endocytosis and ClC‐5‐dependent exocytosis may regulate NHE3‐mediated proximal tubular sodium reabsorption and consequently paracellular calcium reabsorption. The importance of NHE3 dysfunction in determining DD1 phenotype was not investigated in patients with DD1. However, it could be hypothesized that the high rate of hypokalemia among adults patients with DD1 might be due to the increased Na^+^ delivery into the distal tubules and the activation of the renin‐angiotensin system secondary to hypovolaemia may result in metabolic alkalosis, with hypokalemia as a secondary phenomenon. Thus, NHE3 appears to play a crucial role in PT receptor‐mediated endocytosis, suggesting that ClC‐5‐dependent NHE3 dysfunction might be an important factor in the cascade of events leading to the common DD1 phenotype that includes LMWP, hypercalciuria and hypophosphatemia. These observations relate to recent in vivo studies in hypercalciuric humans that implicate the PT as the site of abnormal calcium reabsorption. Thus, the insights from the rare disease DD1 may have implications for the more common phenotype of hypercalciuria and idiopathic calcium stones.

## CONFLICTS OF INTEREST

The authors confirm that there are no conflicts of interest.

## AUTHOR CONTRIBUTIONS

AF, LBL and JL wrote the manuscript, LG conceived and designed figures and contributed to the discussion on the review topics.

## Data Availability

Data sharing is not applicable to this article as no new data were created or analysed in this study.

## References

[1] Edvardsson VO , Goldfarb DS , Lieske JC , et al. Hereditary causes of kidney stones and chronic kidney disease. Pediatr Nephrol. 2013;28:1923‐1942. 23334384 10.1007/s00467-012-2329-z PMC4138059

[2] Wang X , Anglani F , Beara‐Lasic L , et al. Investigators of the rare kidney stone consortium. Glomerular pathology in dent disease and its association with kidney function. Clin J Am Soc Nephrol. 2016;11:2168‐2176. 27697782 10.2215/CJN.03710416 PMC5142066

[3] Scheinman SJ . X‐linked hypercalciuric nephrolithiasis: clinical syndromes and chloride channel mutations. Kidney Int. 1998;53:3‐17. 9452994 10.1046/j.1523-1755.1998.00718.x

[4] Hoopes RR Jr , Hueber PA , Reid RJ Jr , et al. CLCN5 chloride‐channel mutations in six new North American families with X‐linked nephrolithiasis. Kidney Int. 1998;54:698‐705. 9734595 10.1046/j.1523-1755.1998.00061.x

[5] Blanchard A , Curis E , Guyon‐Roger T , et al. Observations of a large Dent disease cohort. Kidney Int. 2016;90:430‐439. 27342959 10.1016/j.kint.2016.04.022

[6] Stölting G , Fischer M , Fahlke C . CLC channel function and dysfunction in health and disease. Front Physiol. 2014;5:378. 25339907 10.3389/fphys.2014.00378 PMC4188032

[7] Novarino G , Weinert S , Rickheit G , Jentsch TJ . Endosomal chloride‐proton exchange rather than chloride conductance is crucial for renal endocytosis. Science. 2010;328:1398‐1401. 20430975 10.1126/science.1188070

[8] Obermuller N , Gretz N , Kriz W , Reilly RF , Witzgall R . The swelling‐activated chloride channel ClC‐2, and the chloride channels ClC‐3 and ClC‐5 are each expressed in distinct subpopulations of renal epithelial cells. J Clin Invest. 1998;101:635‐642. 9449697 10.1172/JCI1496 PMC508607

[9] Sakamoto H , Sado Y , Naito I , et al. Cellular and subcellular immunolocalization of ClC‐5 channel in mouse kidney: colocalization with H+‐ATPase. Am J Physiol. 1999;277:F957‐F965. 10600943 10.1152/ajprenal.1999.277.6.F957

[10] Luyckx VA , Goda FO , Mount DB , et al. Intrarenal and subcellular localization of rat CLC5. Am J Physiol. 1998;275(5):F761‐F769. 9815133 10.1152/ajprenal.1998.275.5.F761

[11] Devuyst O , Christie PT , Courtoy PJ , Beauwens R , Thakker RV . Intra‐renal and subcellular distribution of the human chloride channel, CLC‐5, reveals a pathophysiological basis for Dent's disease. Hum Mol Genet. 1999;8:247‐257. 9931332 10.1093/hmg/8.2.247

[12] Gunther W , Luchow A , Cluzeaud F , Vandewalle A , Jentsch TJ . ClC‐5, the chloride channel mutated in Dent's disease, colocalizes with the proton pump in endocytically active kidney cells. Proc Natl Acad Sci USA. 1998;95:8075‐8080. 9653142 10.1073/pnas.95.14.8075 PMC20931

[13] Pusch M , Ziffarelli G . ClC‐5: Physiological role and biophysical mechanisms. Cell Calcium. 2015;58:57‐66. 25443653 10.1016/j.ceca.2014.09.007

[14] Hara‐Chikuma M , Wang Y , Guggino SE , Guggino WB , Verkman AS . Impaired acidification in early endosomes of ClC‐5 deficient proximal tubule. Biochem Biophys Res Commun. 2005;329:941‐946. 15752747 10.1016/j.bbrc.2005.02.060

[15] Smith AJ , Lippiat JD . Direct endosomal acidification by the outwardly rectifying CLC‐5 Cl(‐)/H(+) exchanger. J Physiol. 2010;588:2033‐2045. 20421284 10.1113/jphysiol.2010.188540 PMC2911210

[16] Wang Y , Cai H , Cebotaru L , et al. ClC‐5: role in endocytosis in the proximal tubule. Am J Physiol Renal Physiol. 2005;289:F850‐F862. 15942052 10.1152/ajprenal.00011.2005

[17] Hryciw DH , Wang Y , Devuyst O , Pollock CA , Poronnik P , Guggino WB . Cofilin interacts with ClC‐5 and regulates albumin uptake in proximal tubule cell lines. J Biol Chem. 2003;278:40169‐40176. 12904289 10.1074/jbc.M307890200

[18] Christensen EI , Devuyst O , Dom G , et al. Loss of chloride channel ClC‐5 impairs endocytosis by defective trafficking of megalin and cubilin in kidney proximal tubules. Proc Natl Acad Sci USA. 2003;100:8472‐8477. 12815097 10.1073/pnas.1432873100 PMC166253

[19] Norden AG , Lapsley M , Igarashi T , et al. Urinary megalin deficiency implicates abnormal tubular endocytic function in Fanconi syndrome. J Am Soc Nephrol. 2002;13:125‐133. 11752029 10.1681/ASN.V131125

[20] Santo Y , Hirai H , Shima M , et al. Examination of megalin in renal tubular epithelium from patients with Dent disease. Pediatr Nephrol. 2004;19(612–5):18. 10.1007/s00467-004-1445-9 15052463

[21] De S , Kuwahara S , Saito A . The endocytic receptor megalin and its associated proteins in proximal tubule epithelial cells. Membranes. 2014;4:333‐355. 25019425 10.3390/membranes4030333 PMC4194038

[22] Sayer JA , Carr G , Pearce SH , Goodship TH , Simmons NL . Disordered calcium crystal handling in antisense CLC‐5‐treated collecting duct cells. Biochem Biophys Res Commun. 2003;300:305‐310. 12504084 10.1016/s0006-291x(02)02837-1

[23] Sayer JA , Carr G , Simmons NL . Calcium phosphate and calcium oxalate crystal handling is dependent upon CLC‐5 expression in mouse collecting duct cells. Biochim Biophys Acta. 2004;1689:83‐90. 15158917 10.1016/j.bbadis.2004.02.007

[24] Carr G , Simmons NL , Sayer JA . Disruption of clc‐5 leads to a redistribution of annexin A2 and promotes calcium crystal agglomeration in collecting duct epithelial cells. Cell Mol Life Sci. 2006;63:367‐377. 16429322 10.1007/s00018-005-5510-8 PMC11136216

[25] Pham PC , Devuyst O , Pham PT , et al. Hypertonicity increases CLC‐5 expression in mouse medullary thick ascending limb cells. Am J Physiol Renal Physiol. 2004;287:F747‐F752. 15161605 10.1152/ajprenal.00229.2003

[26] Bastani B . Immunocytochemical localization of the vacuolar H(+)‐ATPase pump in the kidney. Histol Histopathol. 1997;12:769‐779. 9225160

[27] Wagner CA , Rubio‐Aliaga I , Biber J , Hernando N . Genetic diseases of renal phosphate handling. Nephrol Dial Transplant. 2014;29:iv45‐54. 25165185 10.1093/ndt/gfu217

[28] Blaine J , Chonchol M , Levi M . Renal control of calcium, phosphate, and magnesium homeostasis. Clin J Am Soc Nephrol. 2015;10:1257‐1272. 25287933 10.2215/CJN.09750913 PMC4491294

[29] Moor MB , Bonny O . Ways of calcium reabsorption in the kidney. Am J Physiol Renal Physiol. 2016;310:F1337‐F1350. 27009338 10.1152/ajprenal.00273.2015

[30] Riccardi D , Valenti G . Localization and function of the renal calcium‐sensing receptor. Nat Rev Nephrol. 2016;12:414‐425. 27157444 10.1038/nrneph.2016.59

[31] Capasso G , Geibel PJ , Damiano S , Jaeger P , Richards WG , Geibel JP . The calcium sensing receptor modulates fluid reabsorption and acid secretion in the proximal tubule. Kidney Int. 2013;84:277‐284. 23615500 10.1038/ki.2013.137

[32] Ketchem CJ , Khundmiri SJ , Gaweda AE , et al. Role of Na+/H+ exchanger regulatory factor 1 in forward trafficking of the type IIa Na+‐Pi cotransporter. Am J Physiol Renal Physiol. 2015;309:F109‐F119. 25995109 10.1152/ajprenal.00133.2015 PMC4504931

[33] Forster IC , Hernando N , Biber J , Murer H . Proximal tubular handling of phosphate: A molecular perspective. Kidney Int. 2006;70:1548‐1559. 16955105 10.1038/sj.ki.5001813

[34] Bacic D , Capuano P , Gisler SM , et al. Impaired PTH‐induced endocytotic down‐regulation of the renal type IIa Na+/Pi‐cotransporter in RAP‐deficient mice with reduced megalin expression. Pflugers Arch. 2003;446:475‐484. 12748857 10.1007/s00424-003-1057-4

[35] Weinman EJ , Lederer ED . NHERF‐1 and the regulation of renal phosphate reabsoption: a tale of three hormones. Am J Physiol Renal Physiol. 2012;303:F321‐F327. 22535796 10.1152/ajprenal.00093.2012 PMC3433861

[36] Slattery C , Jenkin KA , Lee A , et al. Na+‐H+ exchanger regulatory factor 1 (NHERF1) PDZ scaffold binds an internal binding site in the scavenger receptor megalin. Cell Physiol Biochem. 2011;27:171‐178. 21325834 10.1159/000325219

[37] Hryciw DH , Jenkin KA , Simcocks AC , Grinfeld E , McAinch AJ , Poronnik P . The interaction between megalin and ClC‐5 is scaffolded by the Na⁺‐H⁺ exchanger regulatory factor 2 (NHERF2) in proximal tubule cells. Int J Biochem Cell Biol. 2012;44:815‐823. 22349218 10.1016/j.biocel.2012.02.007

[38] Hryciw DH , Ekberg J , Ferguson C , et al. Regulation of albumin endocytosis by PSD95/Dlg/ZO‐1 (PDZ) scaffolds. Interaction of Na+‐H+ exchange regulatory factor‐2 with ClC‐5. J Biol Chem. 2006;281:16068‐16077. 16601121 10.1074/jbc.M512559200

[39] Cunningham R , Esmaili A , Brown E , et al. Urine electrolyte, mineral, and protein excretion in NHERF‐2 and NHERF‐1 null mice. Am J Physiol Renal Physiol. 2008;294:F1001‐F1007. 18256311 10.1152/ajprenal.00504.2007

[40] Dusso AS , Brown AJ , Slatopolsky E . Vitamin D. Am J Physiol Renal Physiol. 2005;289:F8‐28. 15951480 10.1152/ajprenal.00336.2004

[41] Chesney RW . Interactions of vitamin D and the proximal tubule. Pediatr Nephrol. 2016;31:7‐14. 25618772 10.1007/s00467-015-3050-5

[42] Wang Y , Zhu J , DeLuca HF . The vitamin D receptor in the proximal renal tubule is a key regulator of serum 1α,25‐dihydroxyvitamin D₃. Am J Physiol Endocrinol Metab. 2015;308:E201‐E205. 25425001 10.1152/ajpendo.00422.2014

[43] Bringhurst FR , Juppner H , Guo J , et al. Cloned, stably expressed parathyroid hormone (PTH)/PTH‐related peptide receptors activate multiple messenger signals and biological responses in LLC‐PK1 kidney cells. Endocrinology. 1993;132:2090‐2098. 8386606 10.1210/endo.132.5.8386606

[44] Zhu Y , He Q , Aydin C , et al. Ablation of the stimulatory G protein α‐subunit in renal proximal tubules leads to parathyroid hormone‐resistance with increased renal Cyp24a1 mRNA abundance and reduced serum 1,25‐Dihydroxyvitamin D. Endocrinology. 2016;157:497‐507. 26671181 10.1210/en.2015-1639 PMC4733111

[45] Silva IV , Blaisdell CJ , Guggino SE , Guggino WB . PTH regulates expression of ClC‐5 chloride channel in the kidney. Am J Physiol Renal Physiol. 2000;278:F238‐F245. 10662728 10.1152/ajprenal.2000.278.2.F238

[46] Piwon N , Günther W , Schwake M , Bösl MR , Jentsch TJ . ClC‐5 Cl‐ ‐channel disruption impairs endocytosis in a mouse model for Dent's disease. Nature. 2000;408:369‐373. 11099045 10.1038/35042597

[47] Günther W , Piwon N , Jentsch TJ . The ClC‐5 chloride channel knock‐out mouse an animal model for Dent's disease. Pflugers Arch. 2003;445:456‐462. 12548389 10.1007/s00424-002-0950-6

[48] Wang SS , Devuyst O , Courtoy PJ , et al. Mice lacking renal chloride channel, CLC‐5, are a model for Dent's disease, a nephrolithiasis disorder associated with defective receptor‐mediated endocytosis. Hum Mol Genet. 2000;9:2937‐2945. 11115837 10.1093/hmg/9.20.2937

[49] Silva IV , Cebotaru V , Wang H , et al. The ClC‐5 knockout mouse model of Dent's disease has renal hypercalciuria and increased bone turnover. J Bone Miner Res. 2003;18:615‐623. 12674322 10.1359/jbmr.2003.18.4.615

[50] Nielsen R , Courtoy PJ , Jacobsen C , et al. Endocytosis provides a major alternative pathway for lysosomal biogenesis in kidney proximal tubular cells. Proc Natl Acad Sci USA. 2007;104:5407‐5412. 17369355 10.1073/pnas.0700330104 PMC1838438

[51] Frymoyer PA , Scheinman SJ , Dunham PB , Jones DB , Hueber P , Schroeder ET . X‐linked recessive nephrolithiasis with renal failure. N Engl J Med. 1991;325:681‐686. 1908057 10.1056/NEJM199109053251003

[52] Luyckx VA , Leclercq B , Dowland LK , Yu AS . Diet‐dependent hypercalciuria in transgenic mice with reduced CLC5 chloride channel expression. Proc Natl Acad Sci USA. 1999;96:12174‐12179. 10518595 10.1073/pnas.96.21.12174 PMC18431

[53] Maritzen T , Rickheit G , Schmitt A , Jentsch TJ . Kidney‐specific upregulation of vitamin D3 target genes in ClC‐5 KO mice. Kidney Int. 2006;70:79‐87. 16672909 10.1038/sj.ki.5000445

[54] Bergsland KJ , Worcester EM , Coe FL . Role of proximal tubule in the hypocalciuric response to thiazide of patients with idiopathic hypercalciuria. Am J Physiol Renal Physiol. 2013;305:F592‐F599. 23720347 10.1152/ajprenal.00116.2013 PMC3891266

[55] Brickman AS , Massry SG , Coburn JW . Changes in serum and urinary calcium during treatment with hydrochlorothiazide: studies on mechanisms. J Clin Invest. 1972;51:945‐954. 4552338 10.1172/JCI106889 PMC302208

[56] Alexander RT , Dimke H . Effect of diuretics on renal tubular transport of calcium and magnesium. Am J Physiol Renal Physiol. 2017;312(6):F998‐1015. 28274923 10.1152/ajprenal.00032.2017

[57] Alexander RT , Dimke H , Cordat E . Proximal tubular NHEs: sodium, protons and calcium? Am J Physiol Renal Physiol. 2013;305:F229‐F236. 23761670 10.1152/ajprenal.00065.2013 PMC4959881

[58] Brett CL , Donowitz M , Rao R . Evolutionary origins of eukaryotic sodium/proton exchangers. Am J Physiol Cell Physiol. 2005;288:C223‐C239. 15643048 10.1152/ajpcell.00360.2004

[59] Pan W , Borovac J , Spicer Z , et al. The epithelial sodium/proton exchanger, NHE3, is necessary for renal and intestinal calcium (re)absorption. Am J Physiol Renal Physiol. 2012;302:F943‐F956. 21937605 10.1152/ajprenal.00504.2010 PMC3330715

[60] Janecke AR , Heinz‐Erian P , Yin J , et al. Reduced sodium/proton exchanger NHE3 activity causes congenital sodium diarrhea. Hum Mol Genet. 2015;24:6614‐6623. 26358773 10.1093/hmg/ddv367 PMC4634371

[61] Janecke AR , Heinz‐Erian P , Müller T . Congenital sodium diarrhea: a form of intractable diarrhea, with a link to inflammatory bowel disease. J Pediatr Gastroenterol Nutr. 2016;63:170‐176. 26835907 10.1097/MPG.0000000000001139

[62] Gallati S . Disease‐modifying genes and monogenic disorders: experience in cystic fibrosis. Appl Clin Genet. 2014;7:133‐146. 25053892 10.2147/TACG.S18675 PMC4104546

[63] Pereira SV , Ribeiro JD , Bertuzzo CS , Marson F . Association of clinical severity of cystic fibrosis with variants in the SLC gene family (SLC6A14, SLC26A9, SLC11A1 and SLC9A3). Gene. 2017;629:117‐126. 28756021 10.1016/j.gene.2017.07.068

[64] Hayashi H , Szaszi K , Grinstein S . Multiple modes of regulation of Na+/H+ exchangers. Ann N Y Acad Sci. 2002;976:248‐258. 12502567 10.1111/j.1749-6632.2002.tb04747.x

[65] Donowitz M , Li X . Regulatory binding partners and complexes of NHE3. Physiol Rev. 2007;87:825‐872. 17615390 10.1152/physrev.00030.2006

[66] Girardi AC , Titan SM , Malnic G , Rebouças NA . Chronic effect of parathyroid hormone on NHE3 expression in rat renal proximal tubules. Kidney Int. 2000;58:1623‐1631. 11012896 10.1046/j.1523-1755.2000.00323.x

[67] Bezerra CN , Girardi AC , Carraro‐Lacroix LR , Rebouças NA . Mechanisms underlying the long‐term regulation of NHE3 by parathyroid hormone. Am J Physiol Renal Physiol. 2008;294:F1232‐F1237. 18322024 10.1152/ajprenal.00025.2007

[68] Ko B . Parathyroid hormone and the regulation of renal tubular calcium transport. Curr Opin Nephrol Hypertens. 2017;26:405‐410. 28614116 10.1097/MNH.0000000000000347

[69] Edwards A , Bonny O . A model of calcium transport and regulation in the proximal tubule. Am J Physiol Renal Physiol. 2018;315:F942‐F953. 29846115 10.1152/ajprenal.00129.2018 PMC6230728

[70] Lin Z , Jin S , Duan X , et al. Chloride channel (Clc)‐5 is necessary for exocytic trafficking of Na+/H+ exchanger 3 (NHE3). J Biol Chem. 2011;286:22833‐22845. 21561868 10.1074/jbc.M111.224998 PMC3123051

[71] Biemesderfer D , Nagy T , DeGray B , Aronson PS . Specific association of megalin and the Na+/H+ exchanger isoform NHE3 in the proximal tubule. J Biol Chem. 1999;274:17518‐17524. 10364184 10.1074/jbc.274.25.17518

[72] Gekle M , Völker K , Mildenberger S , Freudinger R , Shull GE , Wiemann M . NHE3 Na+/H+ exchanger supports proximal tubular protein reabsorption in vivo. Am J Physiol Renal Physiol. 2004;287:F469‐F473. 15113744 10.1152/ajprenal.00059.2004

[73] Okamoto T , Tajima T , Hirayama T , Sasaki S . A patient with Dent disease and features of Bartter syndrome caused by a novel mutation of CLCN5. Eur J Pediatr. 2012;171:401‐404. 21932010 10.1007/s00431-011-1578-3

[74] Bogdanović R , Draaken M , Toromanović A , Đorđević M , Stajić N , Ludwig M . A novel CLCN5 mutation in a boy with Bartter‐like syndrome and partial growth hormone deficiency. Pediatr Nephrol. 2010;25:2363‐2368. 20680351 10.1007/s00467-010-1615-x

[75] Zhu BZ , Li P , Huang JP . [Clinical and genetic analysis of Dent' s disease in 6 Chinese children with low molecular weight proteinuria]. Zhonghua Er Ke Za Zhi. 2010;48:329‐333. 20654030

[76] Besbas N , Ozaltin F , Jeck N , Seyberth H , Ludwig M . CLCN5 mutation (R347X) associated with hypokalaemic metabolic alkalosis in a Turkish child: an unusual presentation of Dent's disease. Nephrol Dial Transplant. 2005;20:1476‐1479. 15814539 10.1093/ndt/gfh799

[77] Seyberth HW . An improved terminology and classification of Bartter‐like syndromes. Nat Clin Pract Nephrol. 2008;4:560‐567. 18695706 10.1038/ncpneph0912

[78] Wojciechowski D , Kovalchuk E , Yu L , et al. Barttin regulates the subcellular localization and posttranslational modification of human Cl(‐)/H(+) antiporter ClC‐5. Front Physiol. 2018;9:1490. 30405442 10.3389/fphys.2018.01490 PMC6206076

